# Hepatocellular adenoma with malignant transformation in male patients with non-cirrhotic livers

**DOI:** 10.1186/s40880-015-0014-x

**Published:** 2015-05-24

**Authors:** Song-Lin An, Li-Ming Wang, Wei-Qi Rong, Fan Wu, Wei Sun, Wei-Bo Yu, Li Feng, Fa-Qiang Liu, Fei Tian, Jian-Xiong Wu

**Affiliations:** Department of Abdominal Surgery, Cancer Institute and Hospital, Chinese Academy of Medical Sciences and Peking Union Medical College, 17 Panjiayuannanli Road, , Chaoyang District Beijing, 100021 P. R. China; Department of Pathology, Cancer Institute and Hospital, Chinese Academy of Medical Sciences and Peking Union Medical College, Beijing, 100021 P. R. China

**Keywords:** Hepatocellular adenoma, Malignant transformation, Hepatectomy

## Abstract

**Introduction:**

Hepatocellular adenomas (HCAs), with a risk of malignant transformation into hepatocellular carcinoma (HCC), classically develop in young women who are taking oral contraceptives. It is now clear that HCAs may also occur in men. However, it is rarely reported that HCAs with malignant transformation occur in male patients with non-cirrhotic livers. This study aimed to characterize the malignancy of HCAs occurring in male patients.

**Methods:**

All patients with HCAs with malignant transformation who underwent hepatectomy at the Cancer Institute and Hospital, Chinese Academy of Medical Sciences and Peking Union Medical College between January 1, 1999 and December 31, 2011 were enrolled in the study. The clinical characteristics as well as radiologic and pathologic data were reviewed.

**Results:**

HCAs with malignant transformation were observed in 5 male patients with non-cirrhotic livers, but not in female patients. The alpha-fetoprotein (AFP) levels were higher in patients with HCAs with malignant transformation than in patients with HCAs without malignant transformation. The diameters of the tumors with malignant transformation were larger than 5 cm in 3 cases and smaller than 5 cm in 2 cases. The 5 patients were all alive without recurrence by the end of the study period. The disease-free survival times of the 5 patients were 26, 48, 69, 69, and 92 months.

**Conclusion:**

Our results indicate that resection would be advised even if the presumptive diagnosis is adenoma smaller than 5 cm in diameter, especially in male patients.

## Background

Hepatocellular adenomas (HCAs) are uncommon and essentially benign tumors of the liver. HCAs occur predominantly, but not exclusively, in young women taking oral contraceptives [1,2].

The annual incidence of HCAs in long-term contraceptive users is 3–4 per 100,000 [1]. These data were, however, recorded in the 1970s from a very limited number of patients and have not been updated since. HCAs have also been described in association with rare conditions such as glycogen storage disease or androgen treatments [3,4].

HCAs in men are rare and usually solitary [5-8]. Patients with HCAs are often overweight with one or several features of the metabolic syndrome. Because of the risk of malignant transformation into hepatocellular carcinoma (HCC), resection of the nodule is recommended even for nodules smaller than 5 cm [9,10].

HCAs are caused by benign proliferation of hepatocytes with high glycogen and fat content, and they lack normal hepatic architecture. HCAs usually present as solitary or multiple tumors that may reach up to 30 cm in diameter [11].

Over the past few decades, several case reports on HCAs supporting the risk of malignancy were published [12-22]. Therefore, it has been accepted that HCA could be transformed into HCC [14-17]. However, the risk of malignant transformation of HCAs cannot be reliably quantified yet. Several studies have shown that approximately 4.2% of patients whose HCAs have been resected had pathologic evidence of HCC within their HCAs [11]. The risk of malignant transformation is associated with the subtype of mutated *β-catenin* and the size of the HCA [11,23]. A study showed that malignant transformation of HCA is unusual for nodules <5 cm [11]. However, detailed molecular studies would be required to prove a direct link between adenoma and carcinoma.

HCCs that developed from HCAs are typically well differentiated without vascular invasion, perforation of visceral peritoneum, or satellite nodules. The alpha-fetoprotein (AFP) level is usually normal and therefore not diagnostically reliable [24,25]. If tumors are large (>5 cm) in diameter, the prognosis is relatively good compared with HCC patients with cirrhosis [25].

In this study, we analyzed the information of HCAs with malignant transformation, comparing with that of HCAs without malignant transformation, and aimed to characterize the malignancy of HCAs occurring in male patients.

## Patients and Methods

### Patient selection

All patients who were diagnosed with HCA at the Cancer Institute and Hospital, Chinese Academy of Medical Sciences and Peking Union Medical College between January 1999 and December 2011 and underwent radical surgical resection were included in this study. Patients with severe cardiorespiratory, liver failure, renal failure, or other malignancies at the time of diagnosis were excluded. The following factors of the patients were assessed: demographic characteristics (age, sex, alcohol intake, tobacco use, and weight), laboratory indicators including hepatits B surface antigen (HBsAg), AFP, platelet (PLT) count, alanine aminotransferase (ALT), albumin (ALB), gamma-glutamyl transferase (GGT), total bilirubin (TBIL), and international normalized ratio (INR) of prothrombin time (PT), tumor parameters (tumor size and number), and operative and perioperative variables (operation time, blood loss, and blood transfusion). This study was approved by the Ethics Committee of the Cancer Hospital, Chinese Academy of Medical Sciences.

### Treatments

The patients with HCA with malignant transformation underwent hepatectomy with margins >1 cm: right lobe irregular hepatectomy or left lateral lobe resection. None of the patients had received any other therapy for HCC, either preoperatively or postoperatively (Table 1). The HCA patients without malignant transformation received irregular hepatectomy with negative margins.

**Table 1:** **Characteristics and laboratory results of patients with hepatocellular adenomas (HCAs) with malignant transformation**

**Case No.**	**Sex**	**Age (years)**	**BMI (kg/m** ^**2**^ **)**	**Sm**	**Al**	**ALT (U/L)**	**GGT`(U/L)**	**TBIL (μmol/L)**	**ALB (g/L)**	**INR**	**HBsAg**	**HBsAb**	**HBcAb**	**AFP (ng/mL)**
1	Male	40	23.99	+	–	35	137	18.3	46.3	0.94	–	–	–	7.56
2	Male	46	26.54	–	–	31	36	17.9	43.8	0.99	–	+	–	4.37
3	Male	51	25.06	–	–	15	22	10.5	39.8	0.92	–	–	–	5.48
4	Male	43	24.86	+	+	22	62	12.4	41.7	0.97	–	–	–	2.24
5	Male	50	29.76	–	+	45	106	6.1	40.5	0.89	–	+	+	3.01

BMI, body mass index; Sm, smoking status; Al, alcohol intake; ALT, alanine aminotransferase; GGT, gamma-glutamyl transferase; TBIL, total bilirubin; ALB, albumin; INR, international normalized ratio of prothrombin time; HBsAg, hepatits B surface antigen; HBsAb, hepatits B surface antibody; HBcAb, hepatits B core antibody; AFP, alpha-fetoprotein. +, positive; −, negative.

### Follow-up

All patients were followed after operation at 3-month intervals for the first year and at 4- to 6-month intervals thereafter. The follow-up program included serum AFP assay, liver function test, abdominal ultrasonography, and chest X-ray examination. Enhanced computed tomography (CT) or magnetic resonance imaging (MRI) was performed every 6 months for surveillance of recurrence. In cases where a suspicious recurrent or metastatic lesion was detected, MRI or hepatic angiography was employed to consolidate the diagnosis.

### Statistical analysis

Statistical analysis was performed by using SPSS 17.0 software. Continuous variables of normal distribution are presented as mean ± standard deviation (SD) and compared by using the independent *t* test. Continuous variables of non-normal distribution are presented as medians and interquartile ranges (IQRs) and were compared by using the Mann–Whitney *U* test. Categorical variables were compared by using Fisher’s exact test. In all cases, statistical significance was defined as *P* < 0.05.

## Results

### Patient characteristics

**Figure 1:**
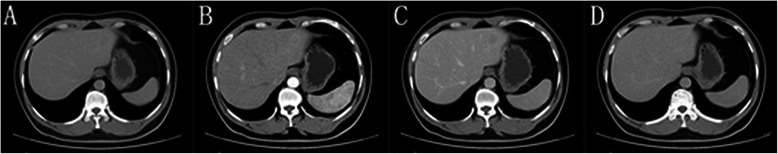
Enhanced computed tomography images show a small mass of approximately 2.5 cm in the largest diameter in the left lateral lobe of the liver. The tumor equidensity in the plain image **(A)**, hyperdensity in the arterial phase **(B)**, and hypodensity in the portal phase **(C)** and the equilibrium phase **(D)**.

**Figure 2:**
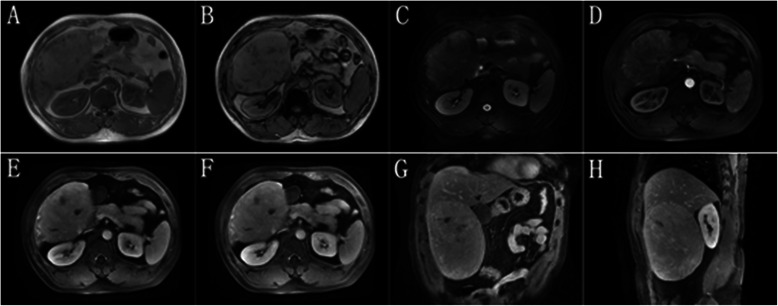
Magnetic resonance images show a large mass involving the right hepatic lobe. The mass with thickened tortuous vessels on the edge shows low signal intensity on T1-weighted dual-echo images **(A and B)**, and slightly long signal intensity on T2-weighted images **(C). D**, in the arterial phase, heterogenous contrast enhancement is noted. **E**, in the portal venous phase, the heterogenous contrast enhancement of the tumor persists. **F**, the enhancement extent of the tumor remains greater than the liver parenchyma in the delayed phase. Coral image **(G)** and sagittal image **(H)** show the tumor and non-tumorous liver simultaneously. The liver is non-cirrhotic.

This study included 5 HCA patients with HCA with malignant transformation and 17 patients with HCA without malignant transformation. The 5 patients with HCA with malignant transformation in this study were all males, aged 40 to 53 years (the median age: 46 years). The clinical presentations of all 5 cases were not very specific; most of them had vague abdominal pain. Two patients had a history of smoking, and 2 had a history of alcohol intake (Table 1). Hepatitis B virus (HBV) infection was not found in any of the patients. However, hepatitis C virus (HCV) infection was found in 1 patient (Case No. 5). Obesity and metabolic dysregulations (diabetes mellitus and hyperlipidemia) were not observed in any of the patients. The serum AFP levels were all ≤ 20 ng/mL. Preoperative biochemical data, including ALT, GGT, TBIL, ALB, and INR of PT, were not noticeably abnormal (Table 1). Of the 5 patients, 3 underwent right lobe irregular hepatectomy and 2 underwent left lateral lobe resection. No ascites were observed, and all patients were in Child-Pugh Class A. Case No. 2 had familial adenomatous polyposis (FAP). The size of the tumors ranged from 2 to 16 cm (median: 10 cm; mean: 8.10 ± 5.88 cm). Tumors smaller than 5 cm in diameter were found in 2 patients (Table 2). Preoperative imaging data were available for all 5 patients: they all underwent CT examinations, and 2 of them also underwent MRI examinations. Three lesions showed radiographic evidence of HCC (Figure 1): 2 with suggestive hepatic angiomyolipoma (Figure 2) and 1 with suggestive hepatocellular adenoma.

**Table 2:** **Management and outcome of 5 HCAs with malignant transformation**

**Case number**	**Tumor location**	**Size of adenoma (cm)**	**Tumor number**	**Operation**	**Membrane invasion**	**Vascular invasion**	**Differentiation**	**Survival (months)**
1	Right lobe	10	Single	Irregular hepatectomy	No	No	Well	92
2	Left lobe	2.5	Single	Left lateral lobe resection	No	Yes^a^	Well	69
3	Left lobe	2	Single	Left lateral lobe resection	No	No	Well	69
4	Right lobe	16	Single	Irregular hepatectomy	No	No	Well	48
5	Right lobe	10	Single	Irregular hepatectomy	No	No	Well	26

^a^Without invasion of the main vascular branches but with microvascular invasion. HCAs, hepatocellular adenomas.

The 17 HCA patients without malignant transformation in this study included 7 males and 10 females, aged 27 to 71 years (the median age: 33 years). All the 17 patients underwent irregular hepatectomy with negative margins. Compared with the HCA patients, the ratio of males to females was higher in patients with HCA with malignant transformation (*P* = 0.040), and they had higher AFP levels (*P* = 0.009) despite being within the normal range. Furthermore, there were no significant differences in other clinicopathologic characteristics between the HCA patients without malignant transformation and the patients with HCA with malignant transformation (Table 3).

**Table 3:** **Comparison of clinical and pathologic characteristics between HCAs and HCAs with malignant transformation**

**Variable**	**HCA group (** ***n*** **= 17)**	**HCC group (** ***n*** **= 5)**	**Statistic**	***P*** **value**
Age (years)			*t* = 0.935	0.361
Mean	33	46		
Range	27–71	40–53		
Sex (cases)				***0.040***
Male	7	5		
Female	10	0		
Alcohol intake (cases)				0.548
Yes	3	2		
No	14	3		
Overweight (cases)				0.274
Yes (BMI ≥ 25 kg/m^2^)	4	3		
No (BMI < 25 kg/m^2^)	13	2		
HBsAg (cases)				1.000
Positive	1	0		
Negative	16	5		
AFP (ng/mL)	1.90 (1.51, 2.99)	4.37 (2.63, 6.52)	*U* = 9	***0.009***
ALT (U/L)	22 (13, 27)	31 (18, 40)	*U* = 26	0.195
GGT (U/L)	23 (13, 44)	62 (29, 122)	*U* = 18	0.055
TBIL (μmol/L)	8.7 (5.8, 9.8)	12.4 (8.3, 18.1)	*U* = 22	0.108
ALB (g/L)	41.3 ± 4.1	42.4 ± 2.6	*t* = 0.571	0.574
PLT (×10^9^/L)	187.2 ± 40.99	227.6 ± 49.04	*t* = 1.857	0.078
INR	1.00 (0.93, 1.03)	0.94 (0.91, 0.98)	*U* = 27.5	0.238
Number of tumors (cases)				1.000
1	14	5		
≥2	3	0		
Tumor diameter (cm)	6.5 (3.5, 9.0)	10.0 (2.3, 13.0)	*U* = 37	0.666

HCA group includes HCAs without malignant transformation; HCC group includes HCAs with malignant transformation. Continuous variables of normal distribution were expressed as mean ± standard deviation and compared by using the independent *t* test. Continuous variables of non-normal distribution were expressed as medians with IQRs in parentheses and compared by using the Mann–Whitney *U* test. Categorical variables were compared by using the Fisher’s exact test.

GGT, gamma-glutamyl transferase; PLT, platelets; INR, international normalized ratio. Other abbreviations as in Table 2.

### Histological characteristics of HCAs with malignant transformation

**Figure 3:**
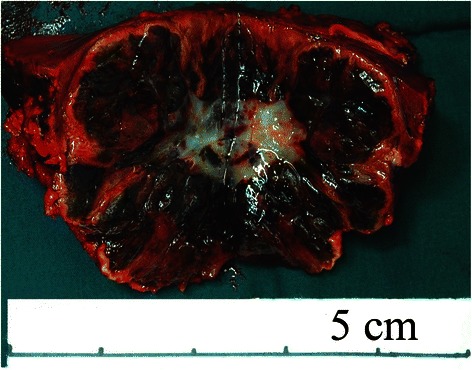
The gross specimen shows a well-demarcated mass in the liver measuring 4.5 cm in diameter. There are congestion and hemorrhage in most of the tumor tissues, and a yellow stellate nodule in the center of the specimen. Uninvolved liver tissues are grossly unremarkable.

**Figure 4:**
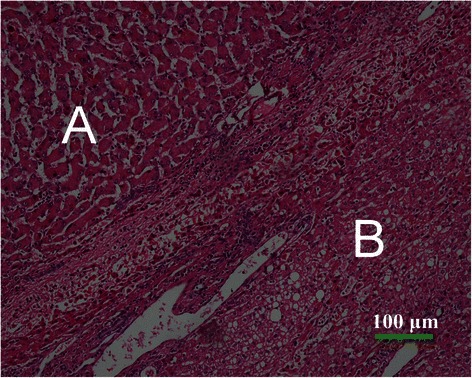
Microscopic findings of hepatocellular adenoma (HCA) with malignant transformation (hematoxylin-eosin staining × 100). On microscopic examination, HCA is composed of uniform benign-appearing hepatocytes **(A)**, and hepatocellular carcinoma (HCC) comprises tumor cells with a high nuclear to cytoplasmic ratio with nuclear irregularities and plates more than two cells in thickness **(B)**.

**Figure 5:**
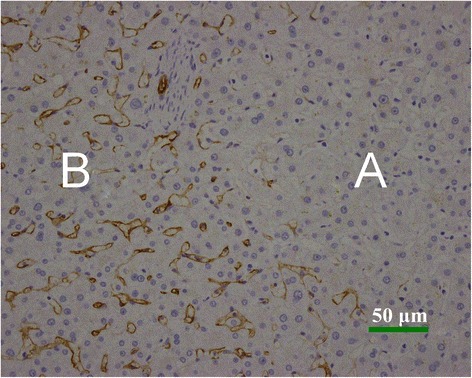
Immunohistochemical analysis of CD34 in HCA and HCC (immunohistochemical staining × 200). The CD34 expression is negative in HCA **(A)**, whereas the positive staining of CD34 is strong and diffused in HCC **(B)**.

By visual examination, the tumors were soft, round, yellow or tan masses and often with areas of necrosis, hemorrhage, and fibrosis. One specimen showed a yellow stellate nodule in the center (Figure 3). The liver background showed mild macrovesicular steatosis in 1 case and mild portal chronic inflammation in 2 cases. However, no significant fibrosis was observed in any of the 5 cases. The histological grades of HCC in the 5 cases of HCAs with malignant transformation were well differentiated. Microvascular invasion was identified in 1 case, and no angiolymphatic invasion was identified in any case. As a typical characteristic of HCC, extensive reticulum loss was observed in all of the carcinomas compared with the background adenomas that retained normal reticulin patterns (Figure 4). The surgical resection margins were negative in all 5 cases. Immunohistochemical staining for CD34 was negative in the adenomas, whereas it was positive within the cancerous areas in all 5 cases (Figure 5).

### Survival

By the end of this study (October 1, 2013), all patients were alive without recurrence. The median follow-up duration was 69 months (range: 26–92 months), and the disease-free survival for the 5 patients with HCA with malignant transformation was 26–92 months (Table 1). The 17 patients with HCAs survived without HCA recurrence or malignant transformation.

## Discussion

In this study, HCAs with malignant transformation were observed in 5 male patients with non-cirrhotic livers but in none of the female patients. AFP levels are higher in HCAs with malignant transformation than in HCAs without malignant transformation. The tumor size was smaller than 5 cm in 2 patients with HCAs with malignant transformation. All 5 patients undergoing hepatectomy were alive without recurrence; the disease-free survival times were 26, 48, 69, 69, and 92 months.

Malignant transformation of HCAs is very unusual because most HCAs are resected at the time of diagnosis. These data might represent an adenoma-carcinoma progression sequence in hepatocarcinogenesis, similar to that observed in colon cancer [26]. The following questions are still being investigated: when HCA develops into malignancy, which factors influence malignant transformation, and what the detailed molecular mechanisms for progression from adenoma to carcinoma are. HCA size, subtype classification, and male gender are major risk factors for malignant transformation. Obesity, diabetes, and combining HCA with FAP are possible risk factors. HBV, HCV, alcohol intake, and tobacco use may also be risk factors.

Most HCAs that become malignant are larger than 5 cm in diameter. Only 4.4% of the cases of malignant transformation were reported in tumors smaller than 5 cm in diameter [11]. However, we have observed tumors with diameters smaller than 5 cm in 2 of 5 cases of HCA, which might be partly because the surgical treatment for HCA is more rigorous in China due to the high prevalence of liver cancer [27]. Therefore, resection should be advised even if the presumptive diagnosis is adenoma with a small tumor, especially in Chinese male patients.

Based on the advancement of molecular and immunohistochemical technologies over the past few years, HCA diagnosis has dramatically changed with the new classification [23,28]. The classification was updated by the World Health Organization, and 4 additional categories are now included: hepatocyte nuclear factor 1α-inactivated, β-catenin–activated, inflammatory, and unclassified [29]. HCAs with mutated β-catenin are more frequently interpreted as borderline lesions between HCA and HCC, and are more frequently associated with the development of unequivocal HCC than other subtypes of HCA [23,28,30].

Several specific risk factors, such as male hormone administration, glycogenosis, familial polyposis, and male gender, have now been identified [31]. The prevalence of malignant HCA is 10 times more frequent in males than in females [11]. A predominance of HCA was not observed in women in China, which could be the result of the birth control policy in China and the use of oral contraceptives in women [27]. According to the birth control policy that became effective in China since 1980, oral contraceptives are commonly used by women before they have their first and usually the only childbirth, and an intrauterine device is then used by most Chinese women as a routine birth control method after the birthchild. In addition, estrogen can protect hepatocytes from malignant transformation. Finally, the proportion of women with heavy alcohol and tobacco use is less than that in men [32]. These reasons might partly explain why women do not show a high prevalence of HCC.

In our institute, all of the patients with HCAs with malignant transformation were males. Therefore, the management of HCA should primarily be based on gender, especially in China.

Malignant transformation of HCA in patients with FAP has been previously reported [33]. In our studies, 1 patient had FAP. Chronic alcohol abuse is known to be a major risk factor for HCC. Alcohol ingestion initiates in the liver and may promote the development of HCC [34]. Tobacco exposure is also a risk factor for HCC. Simultaneous exposure to alcohol and tobacco is expected to promote the development of HCC in an additive and/or synergistic manner. In this study, 1 patient had alcohol abuse, 1 had tobacco use, and 1 had both. Although alcohol intake and tobacco use may promote malignant transformation of HCA, there is no direct evidence to confirm this transformation. Most cases of HCC are attributable to hepatitis virus–related chronic liver disease such as chronic infection by HBV and HCV. Nevertheless, there were only a few reported cases where HCC developed from HCA with HBV infection and none with HCV [35]. However, there was 1 patient with chronic infection of HCV in our study group. It is not clear yet whether HBV or HCV is a risk factor for malignant transformation of HCA. Metabolic syndrome was also reported as an emerging condition associated with the malignant transformation of HCA, particularly in men. However, it is interesting that, besides being male and 1 patient with FAP, patients in this study did not have any metabolic disease or a medication history with steroid hormones. Further investigations are needed for defining risk factors and molecular pathways of HCC from HCA.

Our study is a retrospective study performed at a single center, and further studies at multiple centers are needed to confirm our conclusions. We found that AFP levels were higher in HCAs with malignant transformation than in HCAs, despite being within the normal range. We are not able to explain this finding and its value.

## Conclusions

To summarize, our study suggested that HCCs can arise from HCAs in male patients without cirrhosis who may not have any known risk factors for HCC. Therefore, complete resection is recommended in male patients even if the presumptive diagnosis is HCA in a small size.

## Consent

Written informed consent was obtained from the patient for the publication of this report and any accompanying images.
